# A Review of Translational Research for Targeted Therapy for Metastatic Colorectal Cancer

**DOI:** 10.3390/cancers15051395

**Published:** 2023-02-22

**Authors:** Samantha M. Ruff, Timothy M. Pawlik

**Affiliations:** Department of Surgery, The Ohio State University Wexner Medical Center and James Comprehensive Cancer Center, Columbus, OH 43210, USA

**Keywords:** colorectal cancer, metastases, targeted therapy, KRAS, BRAF, microsatellite instability

## Abstract

**Simple Summary:**

Colorectal cancer is the third most common cause of cancer-related death in the United States with 20% of patients presenting with metastatic disease at time of diagnosis. Metastatic colon cancer is often treated with a combination of surgery, systemic therapy, and/or regional therapy. By utilizing the molecular and pathologic features of the primary tumor we can tailor treatment for patients to try and improve outcomes. Basic science work to elucidate new drug targets, understand mechanisms of evasion, and develop drugs and drug combinations is critical to inform clinical trials and identify novel, effective therapies for metastatic colorectal cancer. This review will focus on targeted therapy in the treatment of metastatic colorectal cancer and how the translational research has advanced the field to improve outcomes for patients.

**Abstract:**

Colorectal cancer is the third most common cause of cancer-related death in the United States, with 20% of patients presenting with metastatic disease at the time of diagnosis. Metastatic colon cancer is often treated with a combination of surgery, systemic therapy (chemotherapy, biologic therapy, immunotherapy), and/or regional therapy (hepatic artery infusion pumps). Utilizing the molecular and pathologic features of the primary tumor to tailor treatment for patients may improve overall survival. Rather than a “one size fits all” approach, a more nuanced treatment plan guided by the unique features of a patient’s tumor and the tumor’s microenvironment can more effectively treat the disease. Basic science work to elucidate new drug targets, understand mechanisms of evasion, and develop drugs and drug combinations is critical to inform clinical trials and identify novel, effective therapies for metastatic colorectal cancer. Through the lens of key targets for metastatic colorectal cancer, this review discusses how work in the basic science lab translates into clinical trials.

## 1. Introduction

Colorectal cancer (CRC) is the third most common cause of cancer mortality in the United States, with 20% of patients presenting with metastatic disease at the time of diagnosis; an additional 25% of patients will develop metastatic disease at some point during the course of their disease [1]. Metastatic CRC (mCRC) is often treated with a combination of surgery, systemic therapy (chemotherapy, biologic therapy, immunotherapy), and/or regional therapy (hepatic artery infusion pumps) [1,2,3]. Despite extensive treatment options, patients with mCRC have a 1-, 3-, and 5-year survival of approximately 70–75%, 30–35%, and 20%, respectively [1].

**Figure 1:**
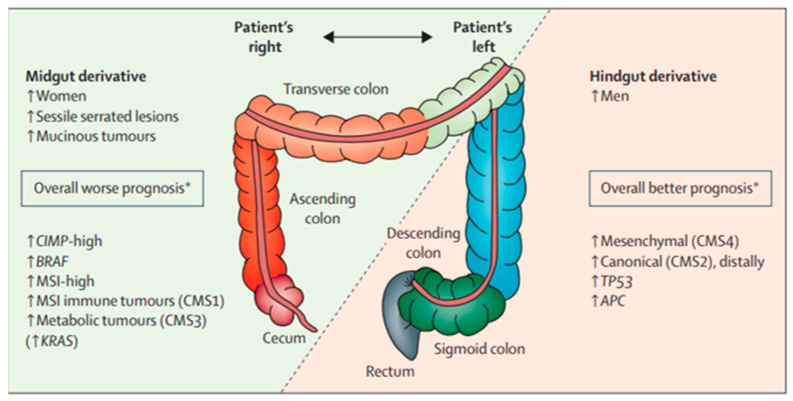
Relationship between clinical, histologic, and molecular differences of colorectal cancer and prognosis [4].

Prognosis for mCRC is often linked directly to the molecular profile of the primary tumor (Figure 1) [4]. The consensus molecular subtype (CMS) classification is based on differences in tumor biology and leverages the heterogeneity of CRC for guidance regarding treatment and prognosis. CMS1 is an upregulation of immune genes and has been associated with microsatellite instability. CMS2 refers to the traditional adenoma–carcinoma pathway with mutations in *APC*, *p53*, and *RAS* and is often characterized by high EGFR expression. CMS3 is due to metabolic dysregulation and higher activity in glutaminolysis and lipidogenesis. CMS4 is related to the activated tissue growth factor β pathway and epithelial–mesenchymal transition [5]. CMS1/CMS3 tumors tend to be right-sided primaries and CMS2/CMS4 tumors tend to be left-sided primaries [5,6,7]. The cancer genome atlas has demonstrated that right-sided tumors display a hypermutated genotype that is largely diploid with prevalent microsatellite instability, while left-sided tumors usually contain *KRAS* mutations and *EGFR/HER2* amplifications [7]. The differences in behavior between left- and right-sided CRC can be traced back to their molecular profile.

Currently, fluoropyrimidine-based systemic chemotherapy is the first-line treatment for mCRC. CRC is, however, a heterogenous disease with numerous genetic origins [1]. As a result, the “one size fits all” approach has evolved to a more personalized approach guided by the unique features of a patient’s tumor and the tumor’s microenvironment. Basic science laboratory findings often focus on identifying the molecular and pathologic features of the primary tumor, as well as the signaling pathways that lead to cancer growth and progression. Understanding the nuances of cancer biology—how cells grow, invade, and establish metastatic niches—is critical to identify targeted therapeutics. Such information can help identify new targets, inform novel targeted therapies, as well as elucidate and overcome mechanisms of evasion/resistance to improve long-term outcomes for patients with colorectal cancer.

While the process of translating what is learned in the laboratory to the clinical setting may appear linear, translational research is more of an evolutionary and cyclical process. The generation, application, and testing of data generated in a laboratory to the clinical setting take enormous effort and time. In fact, all too frequently, success in the laboratory does not necessarily translate to the bedside. We herein review targeted therapy in the treatment of mCRC with an emphasis on how basic laboratory science work combined with clinical trials contributes to improving discovery and implementation of novel therapies.

## 2. Pre-Clinical Models

The complexity of cancer research lies in the heterogeneity of the disease. Even within each type of cancer, there are unique genetic differences and ongoing mutations that result in an ever-changing genomic landscape, allowing for drug evasion and continued tumor growth. Part of the challenge is replicating the genomic heterogeneity of the tumor and its microenvironment in the laboratory setting. The microenvironment must not only account for the interactions among cancer cells, neighboring cells, and immune cells, but also external factors like hypoxia [8]. Pre-clinical models are an avenue for research that eliminates the risk of side effects with the ultimate goal of translating this work into clinical trials. Understanding the strengths and weaknesses of pre-clinical models is key to understanding the challenges of translational work (Table 1).

### 2.1. Cell Lines

Traditionally, two-dimensional cancer cell lines have served as the main research model. For decades, cell lines have allowed researchers to interrogate how genomic aberrations drive tumor progression and help discover potential novel targets for drug therapy [9]. Cell lines are cost-effective and easy to use. More importantly, cell lines represent an unlimited supply of material that provides a consistent sample that can be used to reproduce results [10]. While excellent for early mechanistic work, cell lines exist in a two-dimensional monolayer culture. Unfortunately, this means that cell lines often fail to capture the intricacies of the three-dimensional tumor microenvironment and cell-to-cell interactions. While there have been attempts to use scaffolding to create a three-dimensional model, the results rarely translate accurately into the clinical world [9].

### 2.2. Xenograft Models

Xenograft models are generally created in two ways: through an injection of dissociated human tumor cells or implantation of patient-derived tumor fragments into immune-deficient mice. The injected cell lines can be genetically manipulated, making them ideal for target validation. These models are often cost-effective, and results can be obtained in a few weeks [11]. Unfortunately, these models fail to capture the molecular diversity of tumors and some of the genetic aberrations present are secondary to cell line adaptation to in vitro growth conditions [9]. On the other hand, in patient-derived xenografts, where the tissue undergoes subcutaneous implantation or orthotopic implantation (e.g., CRC implanted into cecum), there is better preservation of the tumor’s genomic characteristics. Studies have demonstrated that the incidence of driver mutations (e.g., KRAS, BRAF) in CRC-patient-derived xenografts mirrors those found in the CRC patient population. In addition, tissue xenografts often have better correlation with clinical outcome data than cell line models [9]. These models require, however, access to surgical specimens and can be labor-intensive, requiring an extended timeline to grow out the xenograft. In addition, the tumor microenvironment in both xenograft models utilizes mouse stromal tissue and does not incorporate an immune system, thereby limiting their utility [9].

### 2.3. Genetically Engineered Mouse Models (GEMMs)

Genetically engineered mouse models (GEMMs) are mice with a modified genome that allows for the natural rise of cancer in the expected organ. This approach has been particularly successful in CRC where clear driver mutations have been identified. The largest advantage of this model is that the mouse has an immune system and more accurately reflects a microenvironment with stromal and immune cell interactions [9]. In addition, the tumor can be followed from early stages and tracked over time, which allows for therapeutic approaches to be explored at different stages of tumor development [11]. Unfortunately, even with the incorporation of a complex tumor microenvironment, there are key biological differences between mouse and human cancers (e.g., tumor suppressor mechanisms). In turn, while this model may be useful for target validation, it is not necessarily predictive of efficacy in the clinical setting [9].

### 2.4. Organoids

A more recent pre-clinical model that has garnered much attention is organoids. Organoids are created by taking a fragment of tumor tissue, dissociating it into a cell suspension, and then placing it on an extracellular matrix scaffold in a culture medium to encourage self-renewal and organized differentiation. The result is a group of three-dimensional clusters that have structural, functional, and molecular similarity to the original tumor [12]. Organoids better represent native tumor tissue and are a superior model for testing cancer therapies. While organoid models have been shown to correlate with clinical outcomes, there are some limitations. Organoids lack stromal tissue, blood vessels, and immune cells, all of which are important to understanding the interaction between the tumor and microenvironment. Some labs are working to co-culture organoid systems to incorporate these elements [13].

## 3. Targets for Metastatic Colorectal Cancer Therapy

### 3.1. Epidermal Growth Factor Receptor (EGFR)

**Figure 2:**
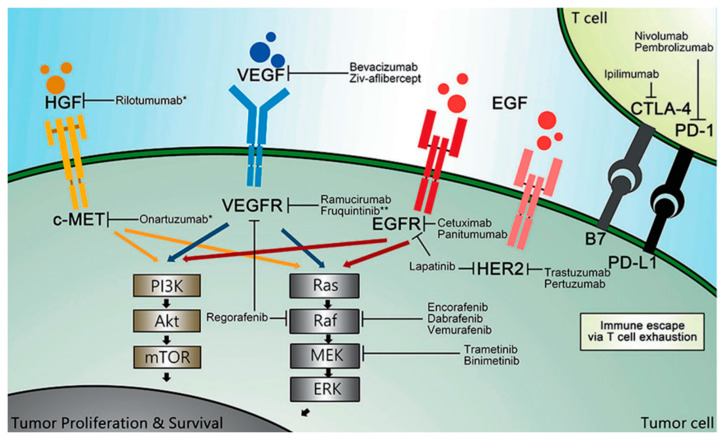
Overview of National Comprehensive Cancer Network (NCCN)-recommended targeted agents. HGF: hepatocyte growth factor; c-MET: mesenchymal–epithelial transition factor; VEGF: vascular endothelial growth factor; VEGFR: vascular endothelial growth factor receptor; EGFR: epidermal growth factor receptor; EGF: epidermal growth factor; HER2: human epidermal growth factor 2; CTLA-4: cytotoxic T lymphocyte-associated antigen 4; PD-1: programmed death-1; PD-L1: programmed death ligand 1; PI3K: phosphoinositide 3-kinase; AKT: protein kinase B, also known as PKB; mTOR: mammalian target of rapamycin; MEK: mitogen-activated protein kinase; ERK: extracellular signal regulated kinase. * These agents have not been recommended by the NCCN. ** This agent has been approved by the National Medical Products Administration of China (NMPA), but not by the United States of America Food and Drug Administration (FDA) [15].

Mechanistic work in the lab can lead to the identification of potential new therapeutic targets. For example, the epidermal growth factor receptor (EGFR) pathway is key in the development and progression of many cancers, including CRC. EGFR is a tyrosine kinase receptor that contains an extracellular ligand-binding region, a single membrane-spanning region, and a cytoplasmic tyrosine-kinase-containing domain. When bound to a ligand, a signaling cascade is started that eventually leads to auto-phosphorylation of tyrosine kinase. Multiple pathways, including the mitogen-activated protein kinase (MAPK) pathway and the phosphatidylinositol 3-kinase (PI3K) protein kinase B pathway, activate transcription factors necessary for cell proliferation, migration, differentiation, and apoptosis (Figure 2). When the regulation of this complex system and its positive/negative feedback loops is altered, it can contribute to malignant transformation and tumor progression [14].

EGFR overexpression on immunohistochemistry (IHC) is associated with tumor progression and poor survival in many cancers. Anywhere from 25 to 82% of CRC tumors are reported to demonstrate EGFR overexpression. However, it is still unclear how EGFR overexpression translates to clinical outcomes in CRC. Some studies have noted that EGFR overexpression is associated with poorly differentiated tumors or reduced survival, while other data have not confirmed these results [16,17,18,19]. In the lab, it has been demonstrated that cell lines with overexpression of EGFR had increased cell proliferation and survival. Further studies using a monoclonal antibody against EGFR in both cell line and xenograft models demonstrated inhibited cell growth [20,21,22].

The BOND trial demonstrated that patients with mCRC who had poor response to single agent irinotecan and were subsequently treated with cetuximab (anti-EGFR monoclonal antibody) had improved progression-free survival [23]. A second trial confirmed that cetuximab resulted in prolonged overall and progression-free survival in patients with mCRC who had previously failed treatment with fluoropyrimidine, irinotecan, and oxaliplatin [24]. In subsequent clinical trials, it was recognized that the addition of cetuximab to first-line standard fluorouracil-based chemotherapy improved outcomes in patients. Importantly, subgroup analysis in the CRYSTAL and OPUS trials demonstrated that anti-EGFR monoclonal antibodies were specifically beneficial for patients with *RAS* wild-type tumors [25,26,27,28,29,30,31]. The recently published FIRE-3 phase III trial randomized patients with *KRAS* exon 2 wild-type mCRC to receive FOLFIRI with either cetuximab or bevacizumab. This study demonstrated that patients who received FOLFIRI with cetuximab versus FOLFIRI with bevacizumab had higher objective response rates (77% vs. 65%, *p* = 0.014, respectively) and longer overall survival (33 vs. 26 months, *p* = 0.011, respectively) [32]. The PARADIGM trial in Japan (NCT02394795) was also recently presented. In this study, the use of panitumumab (EGFR monoclonal antibody) with FOLFIRI was compared with bevacizumab with FOLFIRI in 802 patients who had *RAS* wild-type mCRC. This study demonstrated that patients who received panitumumab over bevacizumab had improved median overall survival (36.2 months versus 31.3 months, *p* = 0.03) [33]. Further examination of EGFR activation and the downstream effects helps to elucidate why the benefit of cetuximab was specific to *RAS* wild-type tumors.

### 3.2. RAS

*RAS* codes for a proto-oncogene in the EGFR signaling pathway that activates the MAPK pathway and leads to increased cell proliferation, migration, invasion, and survival. However, when mutated, RAS proteins can be constitutively activated and lead to uncontrolled cell growth independent of a ligand binding to EGFR [34]. In this scenario, even though cetuximab can bind EGFR, inappropriate downstream activation of the RAS proto-oncogene still occurs, rendering cetuximab ineffective [31]. *RAS* mutation has been identified in 49.7% of all CRC patients [35,36]. Of note, *RAS* mutations are associated with more aggressive tumors, metastatic disease, recurrence, and worse overall survival [37,38,39].

The previously mentioned trials investigating cetuximab (or panitumumab, an alternative EGFR inhibitor) focused on EGFR status. Sub-analysis of patients in these trials demonstrated that the survival benefit associated with EGFR inhibitors was specific to patients with KRAS exon 2 wild-type tumors. In turn, the TAILOR trial, a phase III trial in China, compared FOLFOX with or without cetuximab treatment among patients with mCRC and *RAS* wild-type tumors (*KRAS/NRAS*, exons 2 and 4). Of note, the addition of cetuximab to first-line FOLFOX in patients with *RAS* wild-type mCRC (independent of EGFR status) led to improved progression-free survival, overall survival, and overall response rate. This landmark trial established that cetuximab in combination with FOLFOX should be standard-of-care first-line treatment for patients with RAS wild-type mCRC [40]. The current American Society of Clinical Oncology (ASCO) guidelines recommend anti-EGFR therapy with chemotherapy (FOLFOX or FOLFIRI) as first-line treatment in patients with microsatellite-stable, left-sided *RAS* wild-type mCRC [41].

EGFR inhibitors are not effective in patients with *RAS* mutated mCRC and targeting KRAS has been difficult due to the inability to outcompete GTP’s strong binding affinity to KRAS. KRAS-G12C mutations has been noted in 3% of patients with CRC. Targeting this mutation with an inhibitor has demonstrated significant tumor regression in preclinical patient-derived xenograft models [42]. Sotorasib has recently been introduced as a molecule that specifically and irreversibly inhibits the KRAS protein. Sotorasib covalently binds to the cysteine residue in the P2 pocket and traps the protein in an inactive GDP-bound state, which inhibits downstream signaling. A recent phase II trial examined the use of sotorasib in 62 patients with KRAS-G12C CRC who progressed on FOLFIRI. This study reported an objective response rate of 9.7% [43]. In addition, patients with microsatellite-high CRC have been associated with kinase fusions. These rare but potential targets are currently being investigated and include neurotrophic tropomyosin receptor kinase (NTRK), ROS kinase fusions, RET fusions, neuregulin-1 (NRG1) fusions, and anaplastic lymphoma kinase (ALK) fusions. Multi-kinase inhibitors, including larotrectinib and entrectinib, are currently approved by the Federal Drug Administration for patients with kinase-fusion-positive cancer [44].

### 3.3. BRAF

EGFR stimulation leads to multiple pathways with the potential for mutations at any number of regulatory steps. Identifying mutations along this pathway can help us understand from a mechanistic perspective why some therapies are ineffective and how to overcome drug resistance. An earlier example of this was *RAS* mutation and its effect on the efficacy of EGFR inhibitors. Another example is a mutation in the *BRAF* gene. The *BRAF* gene encodes the BRAF protein kinase in the MAPK signaling cascade that drives cell proliferation, differentiation, migration, survival, and angiogenesis. Almost all *BRAF* mutations are due to a transversion mutation in exon 15 that results in a valine amino acid substitution, V600E. This produces a 10-fold increase in BRAF activity by mimicking regulatory phosphorylation [45].

Approximately 10% of patients with mCRC have a mutation in the *BRAF* gene. Patients are usually female with a right-sided, mucinous cancer and microsatellite instability. Patients with a BRAF mutation often have a poor prognosis (overall survival of about 11 months) and poor response to standard therapies and present with advanced disease [46]. There are two subtypes of *BRAF*-mutant CRC, BM1 and BM2. BM1 is due to activation of the KRAS/AKT pathway, whereas BM2 is due to dysregulation of the cell cycle and cycle checkpoint-related processes [46]. Similar to RAS, BRAF is downstream of EGFR, so there is limited evidence that EGFR inhibitors would result in a clinically significant benefit. In addition, patients with *BRAF* mutations have modest responses to standard cytotoxic chemotherapy. In an effort to improve outcomes, research has focused on BRAF-targeted therapy [47]. However, despite the success of BRAF inhibitors in melanoma, there has been a less impressive response among patients with mCRC. Monotherapy with vemurafenib (BRAF inhibitor) has demonstrated poor results in clinical trials [48,49,50].

When examined in CRC cell lines, BRAF inhibitors only cause partial inhibition of the MAPK pathway due to a suspected feedback loop that re-activates EGFR. This effect has also been demonstrated in CRC tumor xenograft studies [51,52]. Subsequent studies have evaluated the combination of EGFR and BRAF inhibitors in xenograft models in an attempt to increase efficacy. Corcoran et al. reported that EGFR levels were elevated before and after treatment with vemurafenib (BRAF inhibitor), suggesting that EGFR is overactivated in BRAF-mutant CRC cells regardless of BRAF inhibition. These data suggest that even with BRAF inhibition, EGFR activation is still present and transmitted through a different pathway to continue cell proliferation. In concordance with this, BRAF-mutant CRC cell lines express higher levels of EGFR than BRAF-mutant melanoma cell lines, which may explain the difference in the efficacy of BRAF inhibitors in the clinical setting among cancer types. In xenograft models, improved suppression of MAPK signaling and increased tumor regression when treated with a combination of EGFR and BRAF inhibition have been reported [52].

Work in the laboratory setting has led to clinical trials focused on a combination of BRAF and EGFR inhibitors. Compared with other BRAF inhibitors, encorafenib is a BRAF inhibitor with longer pharmacodynamic activity. After earlier clinical trials suggested promising results, the BEACON CRC trial was initiated, which was a phase 3 trial of 665 patients with BRAF V600E-mutated mCRC who had disease progression on other regimens. Patients were randomized to receive encorafenib, binimetinib (MEK inhibitor), and cetuximab (triplet therapy), encorafenib and cetuximab (doublet therapy), or investigators’ choice of cetuximab and irinotecan or cetuximab and FOLFIRI (control group). The median overall survival was 9 months in the triplet cohort, 8.4 months in the doublet cohort, and 5.4 months in the control group. The objective response rate was 26% in the triplet group, 20% in the doublet group, and 2% in the control group [48]. This trial demonstrated for the first time a survival benefit associated with chemotherapy-free targeted treatment in a specific cohort of patients. ASCO guidelines recommend that encorafenib and cetuximab be offered to patients with BRAF 600E-mutated mCRC who have progressed after at least one therapy [41].

### 3.4. Mismatch Repair Mutations (Microsatellite Instability)

Microsatellites are short repeat DNA sequences distributed in both coding and non-coding regions. Due to the repeated structure, these specific DNA sequences are prone to replication errors that are normally repaired through the mismatch repair (MMR) system. Proteins from the MMR system form heterodimers around detected errors, excise the DNA segment, and synthesize a new, corrected strand. The four main MMR genes are *MSH2*, *MLH1*, *MSH6,* and *PMS2*. When deficient MMR (dMMR) is present, these repairs are not carried out, leading to errors in microsatellite sequences. Approximately 15% of patients with colorectal cancer harbor dMMR and subsequent microsatellite instability (MSI) [53]. About two-thirds of these MSI cancers occur sporadically, while the other third are secondary to an inherited germline mutation known as Lynch syndrome [54].

dMMR can be detected through polymerase chain reaction testing for MSI or IHC staining for altered proteins. Patients are classified as MSI-high (MSI-H), MSI-low (MSI-L), or microsatellite-stable (MSS) based on a comparison of five microsatellite markers between healthy and tumor tissue, also known as the Bethesda panel. MSI-H, MSI-L, and MSS are defined by the number of unstable microsatellite markers (more than two out of five, one out of five, or none, respectively). IHC tests for the presence or absence of four MMR proteins (MLH1, MSH2, MSH6, PMS2) to diagnose MSI in a more “indirect” manner [53].

Multiple studies have demonstrated that MSI-H CRC is associated with a more favorable prognosis. MSI-H tumors have been associated with an increase in tumor-infiltrating lymphocytes and more pronounced antitumoral immune response [55]. It is unclear why this relationship exists, although a contributing factor may be that the high mutational load of these tumors results in an increased number of tumor neoantigens [56]. Even though MSI carries a better prognosis, there are some studies that suggest that MSI-H tumors are less responsive to conventional systemic chemotherapy. Rather, immunotherapy has improved outcomes for patients with MSI-H CRC. At this time, three immunotherapy regimens are approved for MSI-H patients: pembrolizumab, nivolumab, and the combination of nivolumab and ipilimumab [1].

The KEYNOTE-164 trial was a phase II trial that evaluated the use of pembrolizumab in 124 patients with previously treated MSI-H mCRC. Patients were divided into two cohorts based on previous regimens. Both cohorts had an overall response rate of 33%, with seven patients having a complete clinical response. With a median follow up of 31 and 24 months, neither cohort reached a median duration of response [57]. There is an ongoing phase III clinical trial (KEYNOTE-177) studying the use of upfront pembrolizumab versus standard chemotherapy for patients with stage IV MSI-H mCRC (NCT02563002). Patients may have received previous adjuvant chemotherapy prior to the study, but therapy must have been received at least 6 months prior to randomization and cannot have been for treatment of metastatic disease.

The phase 2 CHECKMATE-142 study evaluated the use of nivolumab (PD-1 inhibitor) and ipilimumab (CTLA-4 inhibitor) in 45 patients with MSI-H mCRC and no prior treatment for metastatic disease. In this study, there was an overall response rate of 69% and disease control rate of 84%. At 24 months follow up, the progression-free and overall survival was 74% and 79%, respectively. Of note, there was a clinical benefit to treating patients with MSI-H mCRC with nivolumab and low-dose ipilimumab [58].

MSS CRC may be immunologically “cold” with few tumor-infiltrating lymphocytes and therefore unlikely to benefit from immunotherapy. While some preclinical studies have demonstrated that immune checkpoint inhibitors or the combination of an immune checkpoint inhibitor with a biologic may be effective in MSS disease, these findings have not translated into clinical trials [59,60,61]. The KEYNOTE-016 trial evaluated the clinical efficacy of pembrolizumab in patients with MSS and MSI-H metastatic CRC. There was no response noted in the 18 patients with MSS disease, whereas patients with MSI-H disease had an overall response rate of 40% [62]. Pre-clinical work may not always translate into the clinical setting, especially with immunotherapy. Defining and re-creating the human immune microenvironment in the pre-clinical setting is a significant challenge for scientists. One potential approach to overcome this challenge is to investigate mechanisms of resistance in MSS tumors to identify targets that may help to sensitize patients to immune checkpoint inhibitor therapy. For example, a recent study using MSS CRC cell lines and GEMMs demonstrated that IL-17A increases PD-L1 expression and promotes resistance to anti-PD-1 therapy. The investigators reported improved efficacy of anti-PD-1 therapy in MSS CRC murine models by blocking IL-17A [60].

### 3.5. Human Epidermal Growth Factor Receptor 2 (HER2)

HER2 is a tyrosine kinase receptor protein that is a member of the EGFR family. However, unlike other tyrosine kinase receptors, HER2 is triggered by heterodimerization with other ligand-bound receptors (as opposed to binding its own ligand). This results in activation of epithelial cell growth. The incidence of HER2 amplification is 1.3–6% in CRC and represents an important molecular subset. Pertuzumab and trastuzumab combinations have demonstrated some anti-cancer activity in *HER2*-amplified mCRC [63]. The TRIUMPH phase II clinical trial demonstrated that dual HER2 antibodies (pertuzumab and trastuzumab) were effective in patients with HER2-positive mCRC. This study demonstrated an objective response rate of 30% in patients with HER2-positive tissue, 28% in patients with HER2-positive circulating tumor DNA, and 0% in the matched real-world reference population [64]. The MOUNTAINEER trial evaluated the efficacy of trastuzumab and tucatinib in patients with *RAS* wild-type, *HER2*-amplified mCRC and noted an objective response rate of 28.1% with a progression-free survival of 8.2 months [64]. Many clinical trials are still ongoing to better elucidate the most effective anti-HER2 combination therapies.

## 4. Conclusions and Future Directions

While standard cytotoxic chemotherapy has improved outcomes, overall five-year survival for patients with mCRC remains relatively low. mCRC prognosis has been linked to the genetic and molecular profile of the primary tumor. Some findings are secondary to changes in cellular metabolism. Altered cellular metabolism can impact epigenetics in normal cells and cancer cells. The metabolic and subsequent epigenetic changes promote genomic instability, leading to neoplastic transformation. Metabolic dysregulation is a hallmark of cancer that likely contributes and drives other hallmarks of cancer (e.g., genomic instability, mutational burden, proliferative signaling, inflammation, angiogenesis, immune cell evasion) [65]. Targeted therapy directs treatment based on the unique features of each tumor; this includes genetic aberrations and metabolic dysregulation. With the aid of pre-clinical models, research has focused on identifying targets within pathways that drive cancer development and progression. Laboratory findings can be evaluated in subsequent clinical trials to look for correlative success. While transitional research has traditionally been described as “bench to bedside”, it is just as important to work from the “bedside to the bench”. Analysis of clinical trials to identify sub-populations of patients who respond to an intervention and then returning to the lab for better understanding of the underlying mechanism helps to identify which patients will benefit the most from targeted therapy and how drug combinations can overcome drug resistance. Unfortunately, the main challenge with translational research is the pre-clinical models. Most pre-clinical models do not incorporate the genomic complexities of the tumor, immune system, or tumor microenvironment. Targeted therapy has great potential for mCRC, with one example being the success of monoclonal antibodies targeting mutations in the EGFR-activated pathways and immunotherapy for MSI tumors. Continued work to elucidate the molecular subtypes of mCRC, potential targets, and correlation between the bench and bedside is needed to improve outcomes for patients with colon cancer.

## Figures and Tables

**Table 1:** Advantages and disadvantages of pre-clinical models.

Model System	Advantages	Disadvantages
Cell lines	-Cost effective, quick timeline -Easily reproducible and have a consistent sample	-Exist in a 2D monolayer culture and fail to capture the 3D tumor microenvironment
Cell line xenografts	-Cost effective, quick timeline -Cell lines can be genetically manipulated making them ideal for target validation	-Fail to capture molecular diversity of tumors -Genetic aberrations can be due to cell line adaptation to in vitro growth conditions -Mice are immune-deficient so there is no way to study the interactions with the immune system -Tumor microenvironment is made of mouse stromal tissue
Patient derived xenografts	-Preserve tumor’s genomic characteristics -Some correlation between preclinical efficacy and clinical data -Helpful for studying drug resistance mechanisms	-Labor intensive and require access to surgical specimens -Extended timeline needed -Mice are immune-deficient so there is no way to study the interactions with the immune system -Tumor microenvironment is made of mouse stromal tissue
Genetically engineered mouse models	-Tumor arises from the tissue of origin (e.g., colon cancer arises from the colon rather than be implanted into the subcutaneous tissue) -Primary genetic defects are known -Helpful for studying different stages of a disease -Mouse has an immune system	-Extended timeline needed -Key biological differences between mouse and human cancer development (e.g., tumor suppressor mechanisms) -Useful for target validation, but not necessarily predictive of efficacy in clinical setting
Organoids	-3D structural, functional, and molecular similarity to the original tumor -Better represents the native tumor tissue -Correlate with clinical outcomes	-Often lack stromal tissue, blood vessels, and immune cells (although some studies are working to improve this)
